# Disparities in Age at Diabetes Diagnosis Among Asian Americans: Implications for Early Preventive Measures

**DOI:** 10.5888/pcd12.150006

**Published:** 2015-09-10

**Authors:** Monideepa B. Becerra, Benjamin J. Becerra

**Affiliations:** Author Affiliation: Benjamin J. Becerra, School of Allied Health Professions, Loma Linda University, Loma Linda, CA 92350.

## Abstract

We evaluated the association between Asian American ethnicity and age at diagnosis for type 2 diabetes using data from the California Health Interview Survey. Survey-weighted unadjusted and adjusted linear regressions were used to obtain mean estimates of age at diagnosis. In the adjusted regression model, ages at diagnosis were 10.5, 8.7, 8.4, and 4.2 years earlier among South Asian, Vietnamese, Filipino, and Korean populations, respectively, as compared to non-Hispanic whites; no significant difference in age at diagnosis was noted for Chinese and Japanese populations. Recommendations for diabetes screening and preventive measures specific to Asian American populations are warranted.

## Objective

Asian Americans are the fastest-growing population in the United States (1). The literature notes heterogeneity in prevalence of several chronic diseases (2). For example, Asian Indians are twice as likely as Koreans to have heart disease (2), whereas Filipinos and South Asians are more likely than Chinese to be hospitalized for ischemic heart disease (3). Given the differences in the prevalence of cardiovascular disease (CVD) in this diverse group, we hypothesized that specific Asian American subgroups may be at heightened risk for type 2 diabetes, a clinical risk factor for CVD (4).

## Methods

We used the public access adult portion of the 2003–2011 California Health Interview Survey (CHIS). CHIS, a biennial survey using random-digit–dialing, is the largest state health survey and is conducted in several languages, including several Asian languages (5). In this study, we used data for adults who self-identified as Chinese, Filipino, South Asian, Japanese, Korean, or Vietnamese and compared them with data for adults who self-identified as non-Hispanic white, reporting diagnosis of type 2 diabetes. This study was approved by the institutional review board of California State University, San Bernardino.

The primary outcome variable for this study was age at diagnosis of type 2 diabetes, and the primary exposure variable of interest was ethnicity (Asian American subgroup compared with non-Hispanic white). We further included control variables of sex (male, female), education (less than a bachelor’s degree, bachelor’s degree or more), country of birth (United States or foreign), and survey year (2003, 2005, 2007, or 2011).

We weighted all data and used SAS version 9.4 (SAS Institute, Inc), allowing for population estimates. A survey-weighted χ^2^ test of independence was used to assess significant differences in sociodemographic characteristics among Asian Americans and non-Hispanic whites, and survey-weighted multiple linear regression was used to assess age at diagnosis of type 2 diabetes. Relevant interaction terms were further assessed with significance established for analyses (α = .05).

## Results

Most Asian Americans (51.8%) and non-Hispanic whites (54.0%) were female, and a higher percentage for both populations had less than a bachelor’s degree (Table 1). Most Asian Americans were foreign-born (78.5%), but most non-Hispanic whites were US-born (92.0%). Filipinos were the largest Asian American subgroup (37.2%), followed by Chinese (21.9%). South Asians reported the earliest age at diagnosis (44.9 y), followed by Vietnamese (46.7 y), Filipino (47.0 y), Korean (51.2 y), Chinese (52.9 y), and Japanese (55.7 y), compared with an average age at diagnosis of 55.4 years among non-Hispanic whites.

**Table 1:** Study Population (n = 10,445, average annual N = 765,133) Characteristics, California Health Interview Survey, 2003–2011

Characteristic	Asian	Non-Hispanic White
	n (Weighted %)	
**Sex, %**		
Male	734 (48.2)	4,703 (46.0)
Female	747 (51.8)	4,261 (54.0)
**Education^a^, %**		
Less than bachelor’s degree	797 (54.6)	5,924 (69.7)
Bachelor’s degree or more	684 (45.4)	3,040 (30.3)
**Country of birth^a^, %**		
US-born	257 (21.5)	8,321 (92.0)
Foreign-born	1,224 (78.5)	643 (8.0)
**Non-Hispanic white**	—	8,964 (100.0)
**Asian American ethnicity^b^**		
Chinese	334 (21.9)	—
Filipino	269 (37.2)	—
Japanese	187 (11.1)	—
Korean	325 (10.6)	—
South Asian	132 (10.3)	—
Vietnamese	234 (8.9)	—

Abbreviation: —, not applicable.

a *P* < .001.

b Non-Hispanic whites are the reference group.

In the unadjusted model, Filipinos, South Asians, and Vietnamese were diagnosed with type 2 diabetes 5.5 years, 6.9 years, and 5.2 years earlier, respectively, as compared to non-Hispanic whites (Table 2). After adjusting for sex, education level, country of birth, and survey year, age at diagnosis for Filipinos, Koreans, South Asians, and Vietnamese were 8.4 years, 4.2 years, 10.5 years, and 8.7 years earlier, respectively, in comparison to non-Hispanic whites. No significant associations were found between sex and Asian American ethnicity.

**Table 2:** Factors Associated With Age of Onset for Type 2 Diabetes, California Health Interview Survey, 2003–2011

Asian American Racial Subgroups Compared With Non-Hispanic Whites	Unadjusted Mean Estimate, Years (95% CI)	*P* Value	Adjusted Mean Estimate^a^, Years (95% CI)	*P* Value
Chinese	0.5 (−1.5 to 2.6)	.63	−2.5 (−5.2 to 0.2)	.07
Filipino	−5.5 (−8.2 to −2.9)	<.001	−8.4 (−11.6 to −5.1)	<.001
South Asian	−6.9 (−9.8 to −4.1)	<.001	−10.5 (−13.5 to −7.4)	<.001
Japanese	1.2 (−1.9 to 4.3)	.45	0.3 (−2.9 to 3.6)	.84
Korean	−0.7 (−3.0 to 1.5)	.53	−4.2 (−7.0 to −1.3)	.004
Vietnamese	−5.2 (−7.5 to −2.8)	<.001	−8.7 (−11.8 to −5.6)	<.001

Abbreviation: CI, confidence interval.

a Adjusted for sex, education, country of birth, and survey year.

## Discussion

Our study noted that Filipinos, Koreans, South Asians, and Vietnamese adults are likely to be diagnosed with type 2 diabetes significantly earlier than non-Hispanic white adults. Earlier ages at diagnosis indicate the premature presence of clinical risk factors and in turn may explain the increased cardiovascular disease risk noted in some Asian American subgroups (3). Although previous studies have shown a higher prevalence of type 2 diabetes among Asian Indians (2) and Filipinos (6), our study furthers the literature by demonstrating the number of years by which Asian Americans are diagnosed earlier with type 2 diabetes. Younger age at diagnosis could lead to a longer duration of disease, allowing further complications to develop. We also noted that age of diagnosis for Filipinos, Koreans, South Asians, and Vietnamese was considerably younger than the average age of diagnosis for the general United States population (54 years) (7), and that found in our study for non-Hispanic whites (55 years). These disparities indicate that some Asian American populations are at risk of premature chronic diseases.

Such earlier age at diagnosis could be due to differing molecular predisposing factors. For example, Yajnik and colleagues (8) reported, that unlike that in Europeans, a single nucleotide polymorphism in the gene that affects fat mass and obesity was significantly associated with type 2 diabetes among South Asians, even after accounting for body mass index (BMI). Likewise, researchers demonstrated that Filipinas have significantly lower levels of adiponectin, a protein that regulates glucose levels, compared with levels found in whites of similar BMI (9). Similar molecular determinants have been shown in preliminary studies among Vietnamese (10) and Koreans (11). Cumulatively, the literature demonstrates that molecular factors are potentially associated with type 2 diabetes among Asian American subgroups, independent of obesity. These factors could explain the earlier age at diagnosis noted in our study. Guidelines for type 2 diabetes screening among Asian Americans, as proposed by the American Diabetes Association, must go beyond BMI cut-offs (12) and incorporate age cut-offs to ensure early diagnosis and prevention of chronic complications. In addition, our results have immediate implications for California, which spends the least on diabetes prevention measures of all states in the nation (13). Given the burden noted in our study, programs such as those sponsored by the California Heart Disease and Diabetes Prevention Unit of the California Department of Public Health could incorporate age-specific cut-offs for Asian Americans.

Age at diagnosis, however, may not reflect age of onset, so the true burden of the disease may be even more severe among Asian Americans than noted in our study. Future studies addressing age of onset and not just age at diagnosis are critical, because such studies could further identify the groups most at risk, allowing for targeted preventive measures. CHIS does not provide assessment of age of onset, family history, or other sociodemographic variables during the time of diagnosis; lack of such data are a limitation of our study. In addition, our findings are generalizable only to Asian Americans in California; further studies are needed for other regions. Nevertheless, the substantial disparity in age at diagnosis for type 2 diabetes among some Asian American subgroups not only confirms the heterogeneity among the population, but also demonstrates the need to consider developing diabetes screening recommendations that are ethnicity-specific to mitigate the chronic disease burden among this growing population.
